# Dynamic Phase Behavior of Amorphous Solid Dispersions Revealed with *In Situ* Stimulated Raman Scattering Microscopy

**DOI:** 10.1021/acs.molpharmaceut.4c01032

**Published:** 2024-11-19

**Authors:** Teemu Tomberg, Ilona Hämäläinen, Clare J. Strachan, Bert van Veen

**Affiliations:** † Division of Pharmaceutical Chemistry and Technology, Faculty of Pharmacy, University of Helsinki, Helsinki FI-00790, Finland; ‡ Pharmaceutical Sciences, Orion Corporation, Espoo FI-02200, Finland

**Keywords:** amorphous solid dispersion, stimulated Raman scattering microscopy, amorphous−amorphous phase separation, poorly soluble, dissolution, imaging

## Abstract

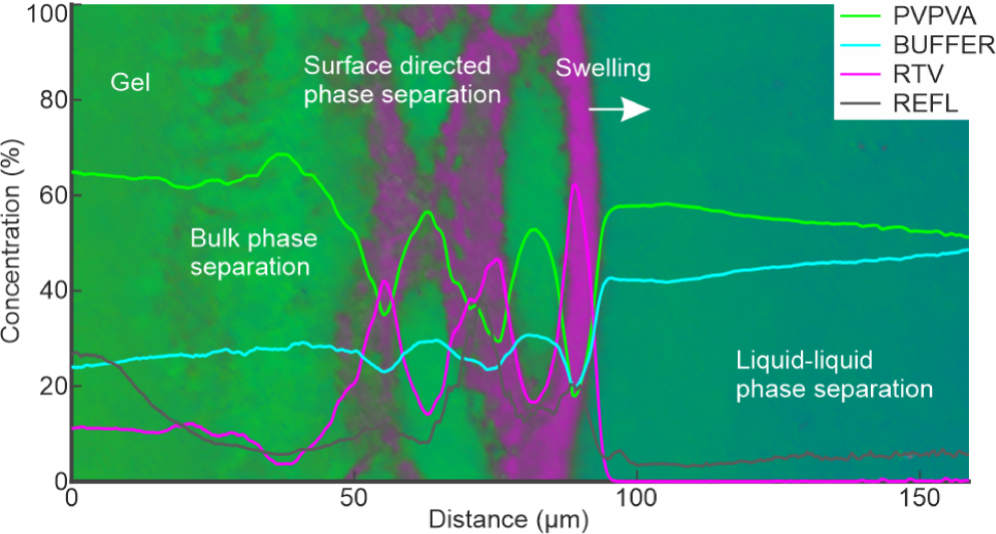

This study reports the application of *in situ* stimulated Raman scattering (SRS) microscopy for real-time chemically specific imaging of dynamic phase phenomena in amorphous solid dispersions (ASDs). Using binary ritonavir and poly(vinylpyrrolidone-vinyl acetate) films with different drug loadings (0–100% w/w) as model systems, we employed SRS microscopy with fast spectral focusing to analyze ASD behavior upon contact with a dissolution medium. Multivariate unmixing of the SRS spectra allowed changes in the distributions of the drug, polymer, and water to be (semi)quantitatively imaged in real time, both in the film and the adjacent dissolution medium. The SRS analyses were further augmented with complementary correlative sum frequency generation and confocal reflection for additional crystallinity and phase sensitivity. In the ASDs with drug loadings of 20, 40, and 60% w/w, the water penetration front within the film, followed by both surface-directed and bulk phase separation in the film, was apparent but differed quantitatively. Additionally, drug-loading and phase-dependent polymer and drug release behavior was imaged, and liquid–liquid phase separation was observed for the 20% drug loading ASD. Overall, SRS microscopy with fast spectral focusing provides quantitative insights into water-induced ASD phase phenomena, with chemical, solid-state, temporal, and spatial resolution. These insights are important for optimal ASD formulation development.

## Introduction

Amorphous solid dispersions (ASDs) represent a key enabling technology to enhance the dissolution of poorly soluble drugs and thus ensure sufficient bioavailability and therapeutic effectiveness.^1,2^ In its most common form, the drug is molecularly and homogeneously dispersed in an amorphous, water-soluble polymer as a glassy solution, at a defined drug-to-polymer ratio. The polymer can provide multiple benefits: during storage, it inhibits crystallization of the drug, while during drug release itself, the polymer enhances both wettability and dissolution of the drug and inhibits crystallization (spring and parachute effect).^3^ Despite successful examples of amorphous solid dispersions on the market, development of such formulations is still highly challenging, not least due to difficulties in understanding and simultaneously optimizing processability, stability during storage, and drug release upon administration.^2^

Besides the selection of a suitable polymer and processing method, the drug-to-polymer ratio is critically important. While a drug loading below the solubility limit ensures a thermodynamically stable ASD (at a given temperature and humidity/water content), higher drug loadings are often required, and instead, kinetic stabilization by the polymer must be relied upon to prevent undesirable physical changes during storage and administration.^3^ Traditionally, inhibition of drug crystallization during storage and drug release has been considered the most important parameters to control during the development of ASDs.^3−5^ However, more recently, amorphous–amorphous phase separation (AAPS) has also been revealed to be critically important. Substantial progress has been made over the past few years into understanding such phase separation, both during storage (as a function of temperature and relative humidity),^6^ and more recently during drug release in aqueous media.^2^

A series of impactful studies in the past few years, in particular by the research groups of Taylor and Sadowksi and their collaborators, have revealed the critically important role of AAPS of ASDs in aqueous media in mediating drug release, particularly as a function of drug loading.^7−11^ The studies have shown that below a threshold drug loading value, typically between 5 and 40% (depending on the drug, polymer, and drug release conditions), the dissolution of the ASD is driven by the polymer solubility in the aqueous media, and drug and polymer release is congruent (i.e., the drug and polymer release concurrently at the same rate). In this scenario, the film may remain as a single phase during drug release, or water-driven AAPS may occur faster than dissolution of the ASD but with the result of the drug-rich phase being disperse (i.e., the drug rich phase exists as droplets/particles within the continuous polymer-rich phase). However, at higher drug loadings, AAPS can occur within and at the surface of the ASD faster than dissolution of the ASD, in which the drug-rich phase becomes the continuous (percolating) phase, and the polymer-rich phase is discrete. In this scenario, the much lower solubility of the amorphous drug itself then dictates release and, to a greater or lesser extent, also the polymer. The consequence of such phase separation can be dramatic, with the drug (and polymer) release often dropping to negligible levels. This has been described as the “falling-off-the-cliff” effect.^7^

*In situ* analysis of the phase separation behavior during drug release is challenging but can be highly informative. Yang and coworkers^7^ probed the role of phase separation and morphology on release behavior of ritonavir-PVPVA ASD tablets in buffer with different drug loadings at 10% intervals. They employed hydrophobic and hydrophilic fluorescent marker molecules and *in situ* confocal fluorescence imaging to indirectly track gel layer development from the matrix–buffer interface in real time, together with the associated drug-polymer phase separation. They observed rapid phase separation into separate drug and polymer-rich domains. At lower drug loadings (10 and 20%), discrete drug-rich domains formed and were associated with polymer-driven release in which the drug and polymer released congruently, while at higher drug loadings (30% and 40%), continuous drug-rich domains were formed at the matrix surface that dramatically inhibited release of both the drug and polymer. Fluorescence imaging with the same marker molecules has been employed to characterize phase separation of an expanding array of APIs and polymers in ASDs, with efforts made to identify the physicochemical properties driving phase separation behavior and associated alterations in drug and polymer dissolution.^8,9,11^

While these studies demonstrate that substantial insights into the phase behavior of ASDs can be gained from the use of fluorescent marker molecules and the instrumentation is highly accessible, direct *in situ* chemically and solid-state specific optical imaging of the API(s) and excipients themselves, at equally good spatial resolution, would have benefits. First, it would allow the sample to be analyzed as is and without any manipulation as such and naturally avoids having to rely on the assumption that the marker molecules associate with the target molecules as predicted. Second, the distribution of numerous components in the system, including water, could potentially be (semi)quantitatively imaged. Finally, a suitable direct imaging technique could also provide solid-state specificity. Attenuated total reflection Fourier transform infrared (ATR-FTIR) macroscopic imaging has been developed by the research group of Kazarian in particular, to quantitively image ASD interactions with dissolution media, including water ingress, drug and polymer dissolution, and drug crystallization in ASDs. They have also coupled the technique with magnetic resonance imaging (MRI) and Raman microscopy (over a much smaller region), to further image water ingress in 3D and drug crystallization, respectively.^5^ While the ATR-FTIR imaging has provided tremendous insights into ASD behavior in dissolution media,^12^ the lateral spatial resolution is at least several micrometers (in contrast to submicron lateral spatial resolution for fluorescence imaging). Furthermore, since the sampling is immediately adjacent to the ASD-ATR crystal interface (within approximately 1 μm), the potential influence of the ATR crystal itself on the ASD behavior, which is not representative of the ASD more generally, needs to be kept in mind. Confocal Raman microscopy has the potential to fulfill the above criteria and, depending on the setup, can offer a spatial resolution comparable to confocal fluorescence microscopy. Recently, Krummnow et al. employed Raman line mapping to quantitatively image water-induced phase separation in amorphous solid dispersions during storage at different humidities, again using ritonavir and PVPVA ASDs as model systems.^6^ Almost a decade earlier, Tres and coworkers^4^ imaged drug loading dependent crystallization of felodipine in PVPVA ASDs in aqueous media with *in situ* confocal Raman microscopy. Both of these studies involved analysis of the ASDs over a time scale of many hours, which allows the sufficient collection of the inherently weak spontaneous Raman scattering signal. These mapping times are too slow to capture fast-paced changes within seconds or minutes that can occur in aqueous media.

Nonlinear optical (NLO) imaging techniques, such as stimulated Raman scattering (SRS) and coherent anti-Stokes Raman scattering (CARS) microscopy, are more advanced Raman-based imaging techniques that excel in rapid three-dimensional visualization of chemical and structural distributions of a sample at a submicrometer level of detail. Sum frequency generation (SFG), on the other hand, can complement coherent Raman microscopy by classifying any bulk structures as noncentrosymmetric crystals or amorphous/centrosymmetric crystals.^13,14^ The advent of modern NLO microscopy occurred in 1999 with the demonstration of Zumbusch et al. of CARS microscopy,^15^ followed by a demonstration of SRS microscopy by Freudiger et al. in 2008.^16^ In the past decade, SRS microscopy has become increasingly popular due to better spectral data and simpler user experience, namely, the lack of a nonresonant background signal and a linear concentration dependence.

CARS microscopy, sometimes augmented with SFG, has been used to image the distribution of API solid-state forms, as well as changes during storage and dissolution.^17−19^ CARS and SRS have also been used to image API and excipient distributions in dosage forms (or intermediates) including tablets,^20−22^ extrudates,^19,23^ films,^24−26^ and API-loaded mesoporous silica microparticles.^27^

The first *in situ* analyses of dosage form changes during drug release involved CARS. Kang et al. imaged the distribution and release of paclitaxel from poly(ethyl-*co*-vinyl acetate) films in an isoporopyl alcohol-phosphate buffered saline solution.^24−26^ CARS has also been used to image theophylline anhydrate release from lipid extrudates in water, as well as simultaneous theophylline monohydrate formation.^19^ More recently, SRS microscopy has been used to image poly(D,L-lactic acid) extrudates as model implant formulations in which particles of the API, entecavir, were distributed. SRS was employed to image the distribution of the two components, as well as the decrease in size and eventual disappearance of individual API particles during drug release in a flow through cell.^23^

In this study, we employ SRS coupled with SFG for the first time for direct *in situ* nondestructive chemically specific imaging of ASD behavior during drug release. Ritonavir (RTV)–polyvinylpyrrolidone vinyl acetate (PVPVA) amorphous solid dispersion films at different drug-to-polymer ratios served as model systems. Different phenomena, including water penetration, amorphous–amorphous phase separation (AAPS), liquid–liquid phase separation (LLPS), and component release, were imaged in real time. Furthermore, the phase separation was understood through the lens of both surface-directed and bulk phase separation.

## Experimental Section

### Materials

**Figure 1:**
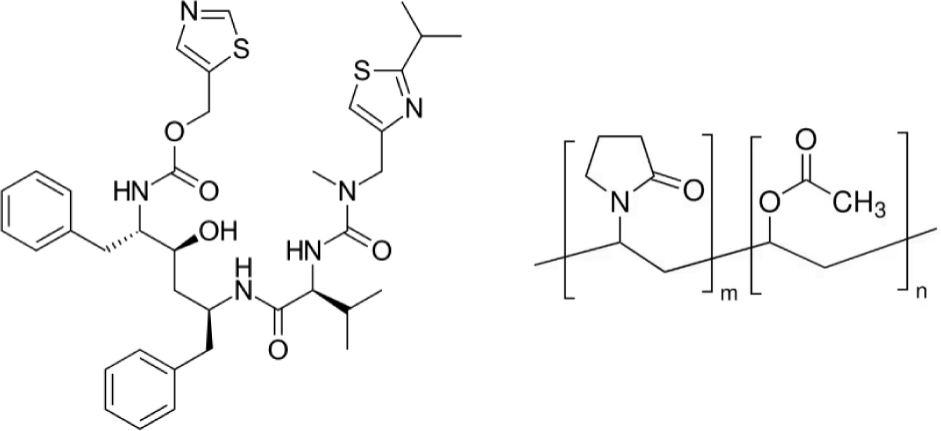
Molecular structures of RTV (left) and PVPVA 64 (right).

Ritonavir (thiazol-5-ylmethyl ((2*S*,3*S*,5*S*)-3-hydroxy-5-((*S*)-2-(3-((2-isopropylthiazol-4-yl)methyl)-3-methylureido)-3-methylbutanamido)-1,6-diphenylhexan-2-yl)carbamate (RTV), Figure 1 (left)) was supplied by BLD Pharm (China) in 99.87% purity. Copovidone (vinylpyrrolidone-vinyl acetate copolymer with a vinylpyrrolidone to vinyl acetate ratio of 6:4 (PVPVA), Figure 1 (right)) was obtained under the brand name Kollidon VA 64 from BASF SE (Ludwigshafen, Germany). Methanol (Emsure for analysis ≥99.9% purity) was supplied by Merck KGaA (Darmstadt, Germany). Phosphate buffer (pH 6.8, 100 mM) was prepared in-house using potassium dihydrogen phosphate (VWR Chemicals BDH, Leuven, Belgium) and sodium hydroxide (Emsure for analysis, Merck KGaA, Darmstadt, Germany).

### Methods

#### Film Preparation

Mixtures of RTV and PVPVA (total 1 g) were prepared at different RTV to PVPVA weight ratios with 20% w/w intervals (0:100, 20:80, 40:60, 60:40, 80:20, and 100:0) and dissolved in 5 mL of methanol (20% w/v solid content) in 14 mL glass vials. The solutions were mixed using a vortex mixer and an ultrasonic bath sonicator (5 min) to ensure complete dissolution. The solutions were then cast onto a glass or aluminum substrate depending on the subsequent analysis technique. The casting was performed in a low humidity environment (20% relative humidity or less) to avoid immediate phase separation in the films. For X-ray powder diffraction (XRPD), polarizing light microscopy (PLM), and spontaneous Raman spectroscopy, 50 μL of each mixture was cast onto 18 × 18 mm square cover glasses (No. 1, EUKITT, ORSAtec). For modulated temperature differential scanning calorimetry (mDSC), the solutions were cast directly into Tzero aluminum pans (TA Instruments, Switzerland). For SRS microscopy, ⌀35 mm glass bottom dishes with a ⌀14 mm microwell and No. 1.5 cover glass (MatTek, USA) were used. A small droplet of the solution was deposited in the center of the microwell. All samples were then immediately placed in a vacuum oven at room temperature for approximately 44 h for residual solvent removal and to prevent water sorption. Subsequently, the samples were stored in a desiccator over silica gel at room temperature until measurement.

#### X-ray Powder Diffraction

Diffractograms of the films were collected using a Rigaku SmartLab diffractometer (Rigaku, Japan) with a Cu Kα radiation source and a HyPix-3000 detector. ASD films were gently scraped with a disposable scalpel, and the scrapings were placed on a low-background sample holder (⌀5 × 0.2 mm) and scanned from 3 to 40° 2θ using a 2° min^–1^ scan rate and a step size of 0.01°.

#### Polarizing Light Microscopy

PLM was performed on the films (film side up) on 18 × 18 mm cover glasses with an Olympus BX50 Polarized Light Microscope (Olympus, Tokyo, Japan) equipped with a Qimaging MicroPublisher 5.0 RTV (Real-Time Viewing) CCD Camera (QImaging, Canada) at 10× and 20× magnification.

#### Differential Scanning Calorimetry

Modulated differential scanning calorimetry (mDSC) was carried out using a Discovery DSC (TA Instruments, New Castle, DE, USA), equipped with a refrigerated cooling system (RCS90). Experiments were performed under a dry nitrogen atmosphere at a flow rate of 50 mL/min, and the temperature was calibrated with pure indium. Tzero aluminum pans (TA Instruments, Switzerland) contained between 2 and 5 mg of each ASD that had previously been cast directly into the pans and vacuum-dried. The pans were sealed with Tzero hermetic lids with pin holes (TA Instruments, USA). Thermal scans of raw materials and ASDs were acquired at a heating rate of 2 °C/min from −20 to 180 °C with a modulation of 1 °C every 60 s.

#### Spontaneous Raman Spectroscopy

Spontaneous Raman spectra of powder raw materials and prepared films with 0% and 100% drug loading were collected with a confocal Raman microscope (NT-MDT Ntegra Spectra, Russia) equipped with a 532 nm laser and 100× objective (Mitutoyo, Japan). The spectral range covered was from 300 to 3500 cm^–1^ with a resolution of approximately 10 cm^–1^ determined by measuring the full width of half-maximum of the Raman peak of silicon at 520.7 cm^–1^. Powdered samples were placed on a microscope slide for the measurement, while the films were measured directly on the cover glasses on which they were prepared. Laser power on the sample was 10 mW, and the acquisition time was adjusted from 1 to 6 s depending on the signal strength.

#### Nonlinear Optical Imaging

Nonlinear optical imaging, including SRS and SFG, was carried out with an in-house built microscope based on an Olympus FV3000 confocal laser scanning microscope (Olympus, Japan) and described in detail elsewhere.^28^ The microscope system features an InSight X3+ (Spectra-Physics, USA) laser and an SF-TRU Timing and Recombination (Newport, USA) for fast hyperspectral SRS imaging.^29^ Laser light was focused on the sample with an Olympus UPLSAPO 60xW1600 objective and collected in the transmission direction with a high NA condenser (Leica, Germany) immersed in the buffer medium if present. The SRS signal was detected with an SRS detection module (APE Angewandte Physik and Elektronik GmbH, Germany), and the SFG signal was detected with a PMT1001 photomultiplier tube module (Thorlabs, Germany), separately. Instrument and experiment control was a combination of commercial FV31-SW software controlling the Olympus microscope frame and an in-house developed LabView (National Instruments, USA)-based user interface controlling the nonlinear optical imaging instrumentation.

**Figure 2:**
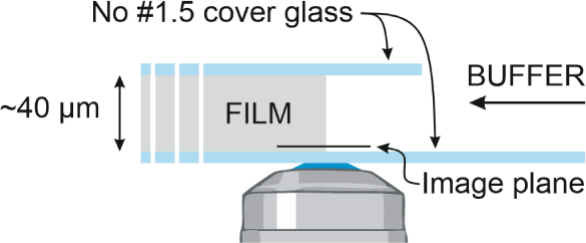
Schematic showing *in situ* imaging setup (not to scale).

A schematic of the sampling setup for *in situ* imaging of the interaction of ASD with the buffer is shown in Figure 2. The samples for SRS imaging were covered with a small, separately cut piece of cover glass (No. 1.5, EUKITT, ORSAtec). The samples were then briefly heated from above using a hot air gun (Hot Air Reworkstation CIF 852-A++, Farnell), allowing the top of the film to soften while gently pressing the cover glass down to adhere it to the film. This preparation method allowed the subsequently added buffer to penetrate only from the side of the film and not the top or bottom such that the interaction of the ASD components perpendicular to the initial ASD-buffer interface could be imaged *in situ*.

#### Reflection Imaging

Phase separation in the ASD films was also correlatively analyzed by simultaneously recording the reflected confocal signal from the same areas of the films using a 640 nm laser and the high sensitivity confocal GaAsP detectors of the FV3000 microscope with the confocal aperture set to 306 μm.

#### Spectral Data Processing

The imaging data were analyzed and visualized with an in-house developed Matlab (MathWorks, USA)-based application. The image sets were first filtered with BM4D, a state-of-the-art unsupervised noise reduction algorithm.^30^ Measurements of pure and dry PVPVA and RTV films and pH 6.8 phosphate buffer were then used to generate reference spectra for non-negative classical least-squares (CLS) unmixing analysis of the data obtained during the *in situ* analyses. For (semi)quantitative analysis, the resulting CLS coefficients (scaling value of each reference spectra in a pixel) were scaled such that in each pixel, the coefficients of the components (PVPVA, RTV, and buffer, depending on the measurement) sum to one (closure). Relative proportions of each component were calibrated using a known concentration location of a measurement, and the calibration was the same for each dissolution experiment.

## Results and Discussion

### Characterization of Starting Materials

#### Solid-State Characterization

**Figure 3:**
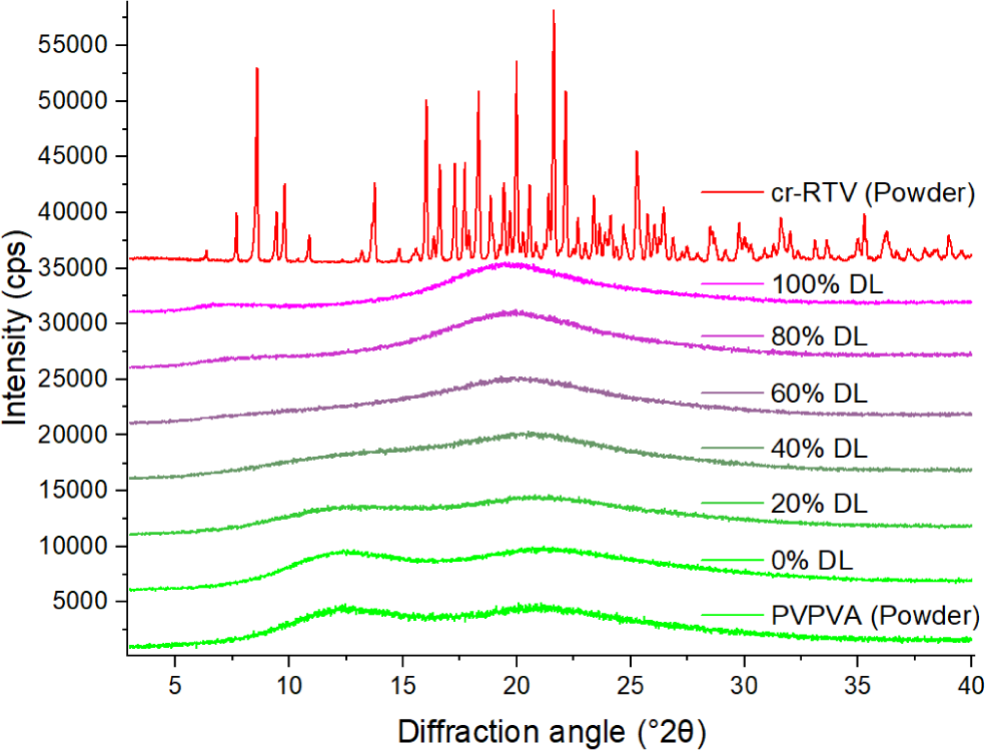
XRPD diffractograms of crystalline RTV and PVPVA powders as received as well as ASD films with different drug loadings (DL, RTV:PVPVA w/w ratios).

**Figure 4:**
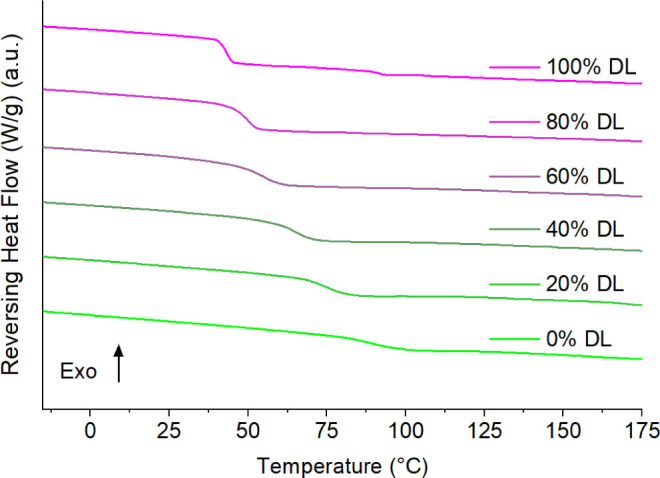
DSC thermograms of ASD films with different drug loadings (DL, RTV:PVPVA w/w ratios).

XRPD of the crystalline RTV starting material was consistent with the stable orthorhombic form II of RTV (Cambridge Structural Database (CSD) RefCODE YIGPIO01, space group P2_1_2_1_2_1_). None of the films exhibited diffraction peaks indicative of crystallinity (Figure 3). Also, according to PLM analyses, the films prepared at all ratios were amorphous, homogeneous single-phase dispersions, and free of trace crystallinity (data not shown). DSC thermograms also suggested the ASDs were single-phase systems, with each exhibiting a single-glass transition temperature (T_g_) progressively decreasing from 89.9 °C (midpoint) for 0% drug loading (100% PVPVA)) to 42.9 °C (midpoint) for 100% drug loading (Figure 4). These T_g_s are slightly lower than the values reported by Krummnow et al.^10^ (at 325.99 and 380.74 K for amorphous RTV and PVPVA, respectively), probably due to different sample preparation conditions and/or measurement parameters.

#### Spontaneous Raman Spectroscopy

**Figure 5:**
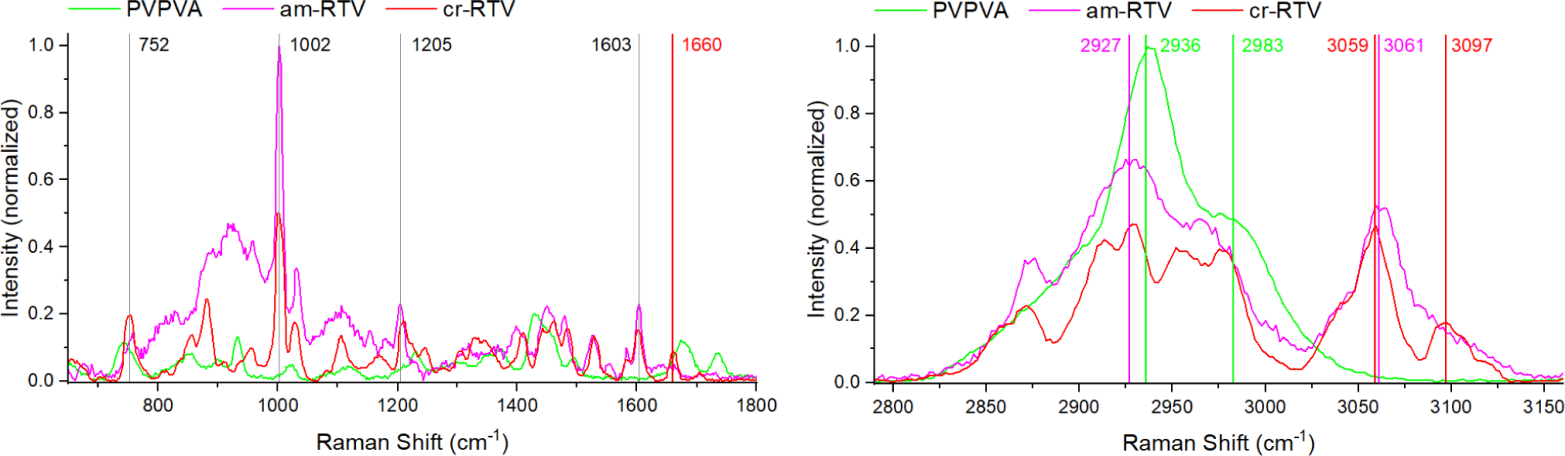
Spontaneous Raman spectra of crystalline RTV (cr-RTV), amorphous RTV (am-RTV), and PVPVA over the spectral ranges 650 cm^–1^ – 1800 and 2790 cm^–1^ – 3160 cm^–1^. Selected Raman shift position characteristics of each of the components are marked.

The Raman spectra of the crystalline and the amorphous pure drug film (Figure 5) are consistent with those previously presented between 600 and 1900 cm^–1^ for form II and the amorphous form of RTV,^31^ including a sharp peak at 1660 cm ^–1^ (C=O stretching) characteristic for form II, which is much smaller and broadened for the amorphous form. We are not aware of published Raman spectra of RTV at higher frequencies, but the CH stretch region was also distinct for the two forms of RTV with peaks at 3059 and 3097 cm ^–1^ for the crystalline form and broader peaks centered at 2927 cm ^–1^ and 3061 cm ^–1^ for the amorphous form. In this region, PVPVA exhibited a peak at 2936 cm^–1^ and a pronounced shoulder at 2983 cm^–1^.

#### Stimulated Raman Scattering

**Figure 6:**
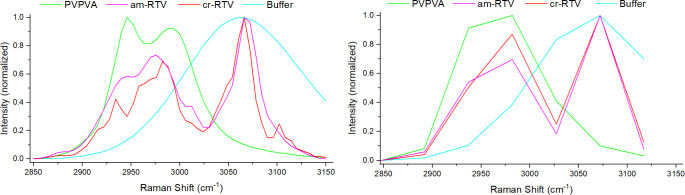
Stimulated Raman spectra of crystalline RTV (cr-RTV), amorphous RTV (am-RTV), PVPVA, and buffer with high (left) and low (right) spectral resolution.

Based on the spontaneous Raman spectra, the spectral region from 2850 to 3150 cm^–1^ was selected for SRS imaging of RTV and PVPVA. Additionally, the phosphate buffer signal (primarily water OH stretching) was recorded in this region. SRS spectra were recorded using two different spectral resolutions: high resolution with a 6 cm ^–1^ spectral step size and 51 spectral points in total (Figure 6, left) and low resolution with a 45 cm^–1^ spectral step size with 7 spectral points in total (Figure 6, right). The first reason for two different settings was optimization of the measurement speed based on requirements of *in situ* dissolution imaging: the lower resolution scan took about 10 s, whereas the higher resolution took about 60 s. Spectral sampling for the low-resolution measurement was designed to be evenly spaced and included the largest peak for each compound. The second reason was the reduction of the exposure to the laser illumination, which effectively reduces the chance for laser-induced photodamage (burning). Any photodamage event we observed was initiated at a single point in the sample, suggesting an absorbing impurity as the cause as opposed to a net heating effect of the sample. Impurities are reported as a common reason for photodamage in solids.^32^ In contrast, we estimate the laser-induced net heating effect to be negligible, based on a recent study involving a combination of SRS and photothermal microscopy.^33^ Faster measurements were used throughout each dissolution experiment, and for the final time point, an image with high spectral resolution was recorded.

At high resolution (Figure 6, left), the spectra are distinct for each component and consistent with the corresponding spontaneous Raman spectra, except for the relative intensities of the spectral features—the SRS intensities toward the center of the spectral range are higher, while those at the edges are diminished, when compared to the spontaneous Raman spectra. This difference is a consequence of the changing pulse overlap employed during spectral focusing (see Figure S1 for a pictorial explanation), causing a Gaussian-like intensity profile multiplying the spectra. The low-resolution spectra (Figure 6, right) are distinct for RTV, PVPVA, and buffer, but cr-RTV and am-RTV are not clearly distinguishable. For this reason, correlative SFG together with the high-resolution spectra was additionally recorded at the end of each experiment to detect any RTV crystallization.

#### Correlative Sum Frequency Generation (SFG)

The three polymorphs of RTV whose crystal structures have been published are the metastable monoclinic form I (space group P2_1_, CSD RefCODE YIGPIO),^34^ stable orthorhombic form II (space group P2_1_2_1_2_1_, CSD RefCODE YIGPIO01),^34^ and metastable monoclinic form III (space group C2, CSD RefCODE YIGPIO05).^31^ All three crystal structures are noncentrosymmetric and are thus capable of sustaining an SFG signal. Consistent with its noncentrosymmetric crystal structure, the crystalline RTV starting material, form II, exhibited SFG (Figure S2), while amorphous RTV, PVPVA, and the buffer did not (data not shown). As such, SFG imaging was subsequently used as an indicator of any RTV crystallization in the films or dissolution media.

### *In Situ* Imaging of Films

#### Pure PVPVA and RTV Films

**Figure 7:**
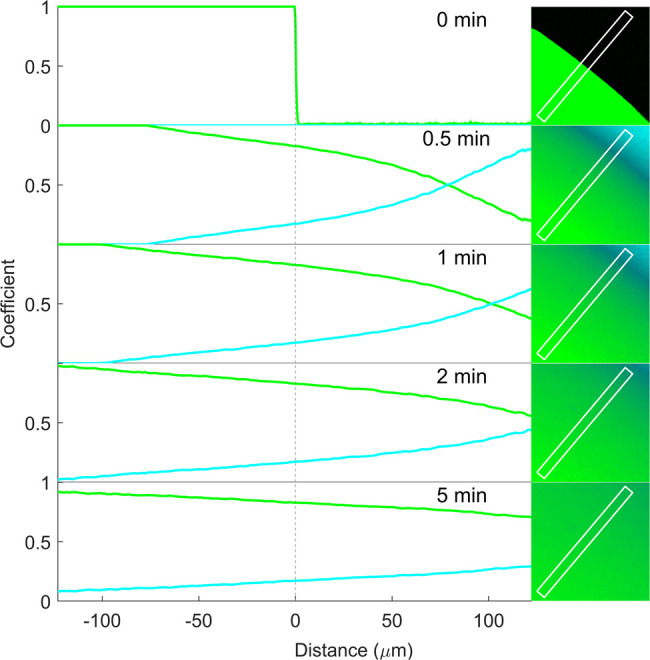
Relative concentration profiles of PVPVA (green) and pH 6.8 phosphate buffer (cyan) before (0 min) and 0.5, 1, 2, and 5 min after buffer addition to pure PVPVA film. Coefficients (representing relative concentration) along the x-axis are the mean (±SD) at each point along the marked rectangular region in the corresponding images to the right. The vertical dotted line at 0 μm represents the film–air interface before buffer addition (0 min). Due to the high degree of content homogeneity at each position along the rectangular region, the standard deviations are not visibly apparent.

We initially imaged the pure PVPVA and RTV films (0% and 100% drug loadings, respectively) as a function of buffer exposure time. Figure 7 shows the time-lapsed images of a neat PVPVA film before and after buffer addition (right), together with (semi)quantitative analyses of the components from the corresponding imaged areas (left). Initial qualitative interpretation of the CLS false color images (right) revealed that the film prior to buffer addition (0 min) had a sharp, clear interface between the homogeneous PVPVA (green) and air (black, due to the sole presence of air). Upon buffer addition (cyan), polymer-buffer mixing was rapid and extensive, and the original interface disappeared already within 30 s. At this time point, the polymer signal (green color) dominated at the greatest depth in the film (bottom left corner of the image), and this gradually gave a way to the intense buffer signal (cyan) on the other side of the original interface (top-right corner of the image). The color became greener and more homogeneous in the imaged area over the next 5 min, indicating increased homogeneity with increasing PVPVA content, consistent with dissolution and diffusion of the polymer in the buffer.

The relative concentration distributions of PVPVA and buffer (or water) at each time point were analyzed (semi)quantitatively using line plots (Figure 7, left). The average normalized CLS regression coefficients of the PVPVA and buffer signals at each position along the length of a rectangle normal to, and crossing over, the initial film–air interface are presented (rectangles shown in the corresponding images on the right Figure 7). As expected, before buffer addition, the PVPVA coefficients within the film are consistently at unity (representing pure PVPVA signal) and then immediately drop to 0 at the film–air interface and remain at 0 on the other side of the interface (due to the presence of air and absence of SRS signal). Within 30 s of buffer addition, the coefficients of both the PVPVA and buffer are both strongly represented either side of the original film–air interface, revealing substantial diffusion and mixing of the two components. Analysis of the buffer coefficients reveals that the waterfront had penetrated approximately 76 and 100 μm into the film after 0.5 and 1 min, respectively. The polymer concentration gradually decreases, and water concentration increases at increasingly positive values on the x-axis, across the field of view, revealing continuous dissolution and diffusion of the polymer into the buffer and *vice versa*. These concentration gradients gradually decrease for both components as a function of time, revealing an increasingly homogeneous PVPVA-polymer solution across the imaged area. There are no abrupt changes in coefficient values at any point, and as such, there is no evidence of the presence of a defined gel–solution interface.

**Figure 8:**
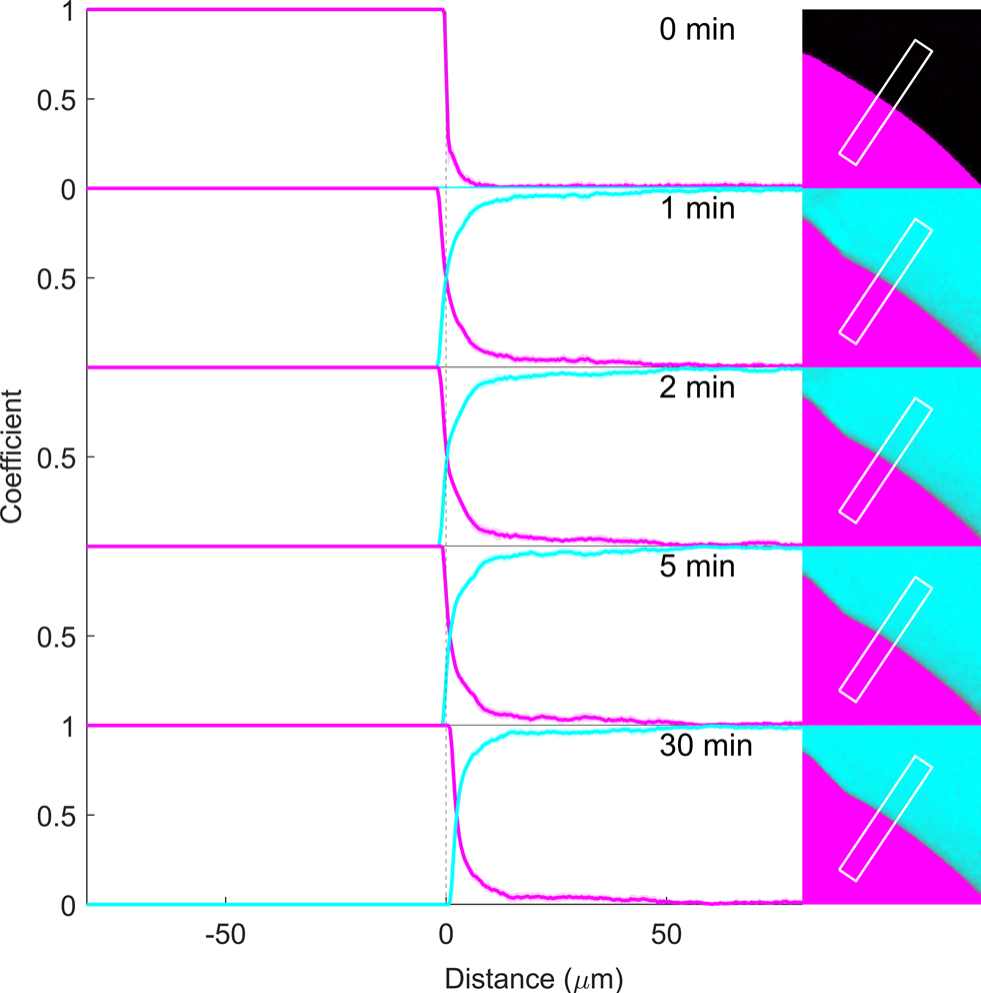
Relative concentration profiles of RTV (magenta) and pH 6.8 phosphate buffer (cyan) before (0 min) and 1, 2, 5, and 30 min after buffer addition to pure RTV film. Coefficients (representing relative concentration) along the x-axis are the mean (±SD) at each point along the marked rectangular region in the corresponding images to the right. The vertical dotted line at 0 μm represents the film–air interface before buffer addition (0 min) and at 1 min (because the sample was moved after the first image at 0 min due to sample burning). Due to the high degree of content homogeneity at each position along the rectangular region, the standard deviations are not visibly apparent.

The equivalent 100% RTV film time-lapse images are shown in Figure 8. The dry film (0 min) was evenly magenta, and there was a sharp matrix–buffer interface, in a similar fashion to that observed for the polymer film. After addition of the buffer, in stark contrast to the PVPVA film, the RTV film appeared largely inert for the duration of the experiment, with the interface remaining at virtually the same position (now representing the RTV–buffer interface). The coefficient values of RTV and buffer were almost completely separate (most of the overlap can be attributed to an uneven interface, already evident from the RTV coefficient values at 0 min) and essentially unchanged for the entire imaging time (up to 30 min), indicating minimal mixing and dissolution of RTV. A very slight shift of the interface to the right at the latter time points suggests some very limited RTV film swelling (Figure 8). Correlative SFG analysis after 30 min additionally confirmed that the RTV remained amorphous (as evidenced through the absence of SFG signal, data not shown), consistent with previously published analyses of amorphous RTV after immersion in pH 6.8 buffer, as determined by *ex situ* Fourier transform infrared (FTIR) spectroscopy and scanning electron microscopy (SEM).^35^

As would be expected for both the single-component films without the possibility for drug-polymer phase separation, the coefficients at each position along the x-axis have negligible standard deviations (not visible), across the entire analyzed area (in Figures 7 and 8, for pure PVPVA and RTV films, respectively).

#### RTV and PVPVA Matrix Films

Matrix films with drug loadings from 20 to 80% were imaged *in situ* in the same manner as the single-component films. However, the chemically specific SRS imaging of these matrices was additionally complemented with correlative confocal reflection images, since the intensity and direction of elastic light scattering are sensitive to phase separation in multicomponent amorphous materials.^36^

An absence of the SFG signal in all samples (correlative SFG analysis was performed before and at the end of the *in situ* SRS analyses) confirmed the absence of any crystallization (data not shown) at all drug loadings, consistent with previous analyses of RTV-PVPVA ASD films exposed to pH 6.8 buffer using ATR-FTIR spectroscopy.^35^

#### 20% Drug Loading

**Figure 9:**
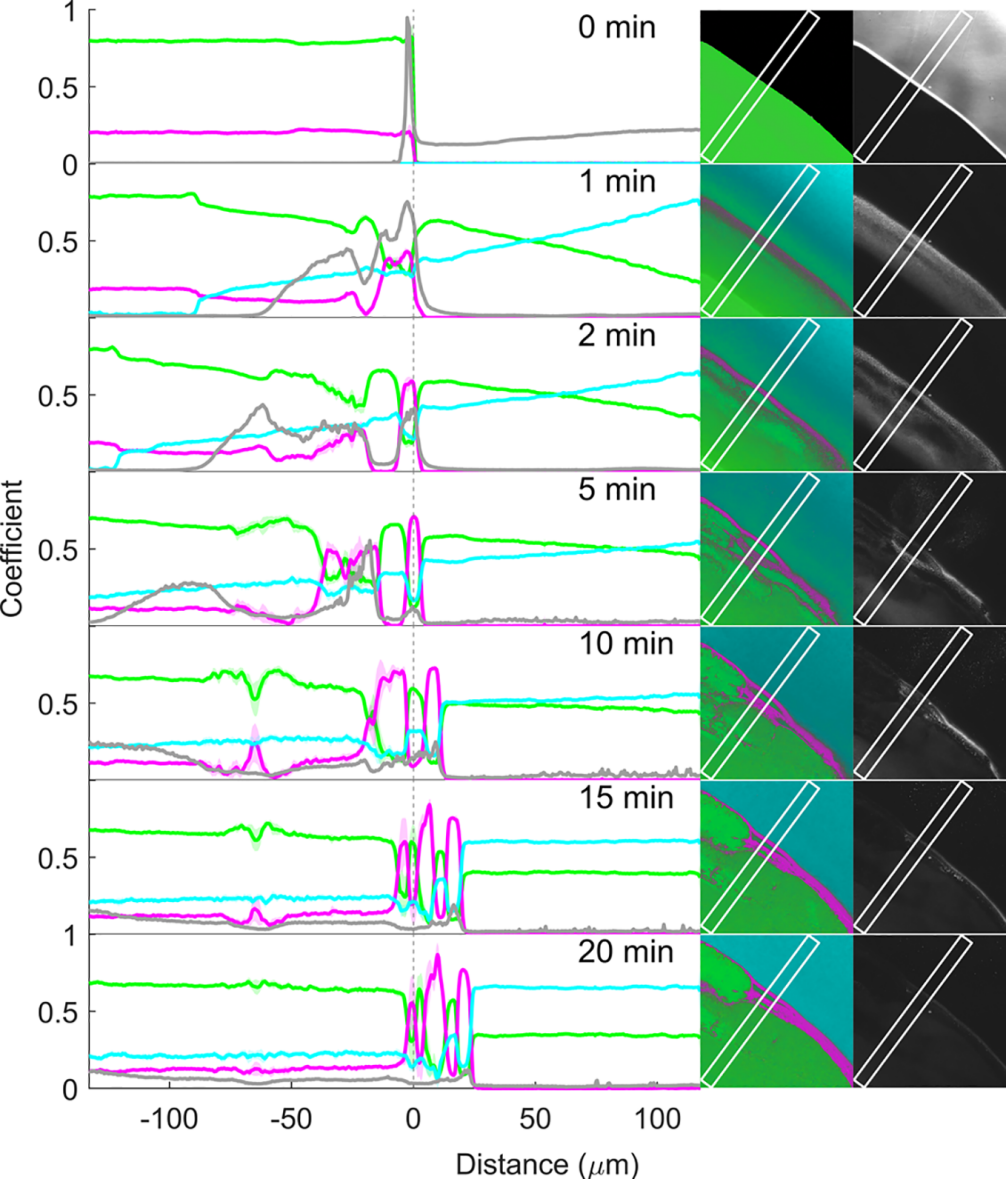
Coefficient (representing relative concentration) profiles of RTV (magenta), PVPVA (green), and pH 6.8 phosphate buffer (cyan), as well as confocal reflection intensity (gray), before (0 min) and 1, 2, 5, 10, 15, and 20 min after buffer addition to the matrix film with 20% drug loading. The coefficients along the x-axis are the mean (±SD for RTV, PVPVA, and buffer) at each point along the marked rectangular region in the corresponding images to the right. The vertical dotted line at 0 μm represents the film–air interface before buffer addition (0 min).

False-color SRS images of a film with 20% drug loading, together with the correlative grayscale confocal reflection images of exactly the same areas, are presented in Figure 9 (right). At each time point, these images are complemented in the same figure with a coefficient plot on the left side of the figure, containing the chemically specific SRS CLS coefficients (representing drug, polymer, and buffer), as well as the correlative reflection coefficients (from exactly the same rectangular area). In the interpretation of the data in Figure 9, first, the chemically specific SRS data are considered, followed by the correlative confocal reflection data.

The false-color SRS image of the dry film (Figure 9, 0 min) was an almost uniform shade of green, consistent with a single-phase homogeneous film rich in PVPVA, and the film–air interface was sharp. The SRS coefficients measured from the rectangle in the image together with minimal standard deviations (not visible) are consistent with a homogeneous distribution of 20% drug and 80% polymer across the film.

Upon buffer addition, the buffer (or water) signal in cyan color is visible toward the top right-hand corner of the images. Water penetration into the matrix resulted in an interface that is visible as a discrete change in shade of green toward the bottom left-hand corner of the SRS images at approximately 87 and 121 μm from the original interface at 1 and 2 min, respectively. The coefficient plot reveals that this represents the penetration of the water front into the film and therefore the glass–gel interface. At this interface, there is a discrete jump of water content from an undetectable level on the glassy side to about 12% water content on the gel side, while the RTV and PVPVA coefficients correspondingly drop by approximately 5% and 7%, respectively. At later time points, this interface penetrated further into the film beyond the field of view. The water penetration into the film and gel formation cause the film to gradually swell, with the matrix–buffer interface expanding into the buffer region by approximately 23 μm at the 20 min time point.

In the gel phase and dissolution medium, the water concentration continues to overall trend upward and the polymer concentration trend downward along the x-axis, for all time points up until and including 10 min, consistent with water penetration into the film and polymer dissolution into the buffer. However, there are strong concentration fluctuations in these upward and downward trends along the x-axis for the buffer and polymer, respectively. Furthermore, the RTV concentration coefficient shows opposing fluctuations in comparison to the polymer. These fluctuations in the gel are evidence of water-induced drug-polymer phase separation.

Already within 1 min of buffer exposure, drug-polymer phase separation has occurred, as evidenced in the false-color SRS images (Figure 9, second column from the right) as drug-rich domains (pink) and polymer-rich domains (green). This is most visible in the false-color image as a thin magenta layer, along the location of the original film–air interface. Immediately to the right of the drug-enriched interface, the polymer coefficients peak and gradually decrease to the right, deeper into the dissolution medium. Thus, as the hydrophilic and water-soluble polymer at the film surface preferentially associates with the buffer by dissolving and diffusing deeper into the dissolution medium, it leaves behind the hydrophobic drug which self-associates, enriches, and forms a new interface matrix in the form of a drug-enriched layer.

Inspection of the corresponding coefficient plot reveals that this drug-enriched layer at 1 min is approximately 10 μm wide and in fact features two drug concentration maxima with a small drug concentration dip in between. Another, less intense, drug maximum is present at the x-axis position of approximately −26 μm. After 2 min, phase separation has intensified—the coefficients reveal sharper concentration differences between the drug-rich and polymer-rich layers. Later time points feature more layers of phase separation parallel to the original film–buffer interface. According to the coefficient plots, both the polymer and buffer coefficients consistently decrease together at the same positions where the drug coefficient increases. This can be attributed to the water preferably associated with the hydrophilic polymer over the hydrophobic drug. This finding is in line with observations of Krummnow et al. (2023),^6^ who used spontaneous Raman line-mapping microscopy to quantitatively image water-induced RTV and PVPVA phase separation in 20% and 25% drug-loaded ASDs over several days at 94% relative humidity.

The false-color images in Figure 9 also show that the water-induced surface-directed phase separation of drug and polymer (and water) within the ASD does not develop in a uniform manner parallel to the original film buffer. This suggests that the process is highly sensitive to experimental conditions and presumably is also partially stochastic in nature. Nevertheless, the observations are consistent with the phenomenon of surface-directed spinodal decomposition, first experimentally reported by Jones et al. (1991)^37^ for unstable polymer blends in which one of the components has a preferential attraction to the surface. They observed composition wave vectors normal to and propagating inward from the interface, maintaining coherence for several wavelengths.

In addition to surface-oriented phase separation, close inspection of the SRS false-color images (Figure 9) reveals a second form of phase separation within the films, namely water-induced bulk phase separation, which has previously been modeled in pharmaceutical PVPVA ASDs in buffer.^6,8,10^ At the 20% drug loading, this manifests as the development of nonsurface-directed drug-rich (magenta) droplets within a continuous polymer-rich (green) network, consistent with previous indirect observation with confocal fluorescence microscopy involving fluorescent dyes,^7,8,35^ as well as thermodynamic perturbed-chain associating fluid theory (PC-SAFT) modeling.^8,10^ At 1 min, this is already observed in the SRS false-color images on the microscale, with the phase separation appearing to extend deeper and deeper into the matrix after longer buffer exposure times. This can also be observed to some extent at 1 min as very fine oscillations in the polymer and drug coefficients between −50 μm and the surface-directed phase separation, and, as the domain sizes and concentration gradients grow (from 2 min onward), also as increased coefficient standard deviations.

Since phase separation in both liquid and gel phases has been extensively studied with various light scattering techniques, such as dynamic light scattering,^35^ we further characterized the phase behavior in the gel phase of the film, as well as the dissolution medium, by employing correlative confocal reflection imaging with a 640 nm laser. While more advanced light scattering techniques can provide more comprehensive information, such as particle size on the nano- to microscale and density,^35,38−42^ confocal reflection imaging provides a simple approach to visualize the onset and propagation of phase separation (without chemical specificity) beyond the resolution limit of chemically specific SRS imaging. The resulting correlative reflection images are presented in grayscale in Figure 9 (far right column, representing exactly the same sample area as the false-color SRS images). Prior to buffer exposure, there is no significant reflection signal from the film (except for at the edge of the film), consistent with a single-phase film (outside the film area, the reflection signal from the glass/air interface disappears once the buffer is added). From 1 min onward, the films exhibit a reflection response visible as gray/white regions in the grayscale images in Figure 9 (right), indicative of nano- and microscale phase separation.

Comparison of the reflection and SRS coefficient plots (Figure 9, left) provides more detailed insight into phase separation in the gel as a function of film depth (negative x-position). In these plots, the reflection intensity from exactly the same positions as the SRS drug, polymer, and buffer coefficients are presented (gray line). The reflection intensity distribution evolves over time. The initial onset of reflection intensity increase appears in the gel phase at approximately −60 μm, −84 μm, and −128 μm at 1, 2, and 5 min, respectively (Figure 9). The increased reflection intensities appear as a “wave” that gradually progresses deeper into the gel phase of the film and represent the first sign of nano- (and then micro-) phase separation. This phase separation front trails the water front (glass–gel interface) by about 27 and 37 μm at 1 and 2 min, respectively. The elevated reflection signal appears earlier (deeper) in the gel phase than any chemically specific SRS evidence of phase separation. This is because elastic scattering of light already occurs with nanoscale phase separation below the applied sampling resolution of the SRS microscope in this experiment (approximated as three times the pixel size of 414 nm). Once the phase separation domain size has increased and is clearly evident with SRS, the elastic reflection signal intensity decreases. The minimum phase-separated domain size required to generate a detectable confocal reflection signal is not defined but is likely on the order of tens of nanometers. As such, correlative nonchemically specific confocal reflection highly complements the chemically specific SRS for *in situ* imaging of water-induced phase separation in ASD films. In Figure S4, a graphical representation of the evolution of three phenomena discussed above, namely, the water penetration front (glass–gel interface, detected with SRS), phase separation front (detected with confocal reflection imaging), and matrix swelling (matrix–dissolution medium interface, detected with SRS) is presented (for the 20% drug loading, as well as higher drug loadings (*vida infra*)).

Liquid–liquid phase separation (LLPS), resulting in the presence of drug-rich droplets within the dissolution medium, has been widely reported for ASDs.^38^ Upon close inspection of the SRS images (Figure 9), a very few drug-rich droplets within the buffer-rich phase are visible from 5 min onward (as magenta dots, highlighted with zoom in Figure S3). In this example, these are not of sufficient quantity and size to be represented as fluctuations in the drug coefficient line plot (with coefficient values remaining close to 0 throughout the buffer-rich phase). However, strong (nonchemically specific) evidence of nanodroplet formation in the buffer-rich phase (below the spatial resolution of the SRS microscope) is provided by the correlative confocal reflection images, in which localized reflections in the buffer-rich phase (white dots) are visible from 5 min onward, together with clear oscillations in the reflection coefficients (Figure 9). A video of the movement of these nanodroplets is also present in Video S1. Furthermore, in a separate example (*vide infra*) of a 20% drug loading film (replicate sample) after 30 min of buffer exposure, more pronounced LLPS with larger droplet sizes is clearly visible with SRS depth profiling (Figure 14).

**Figure 10:**
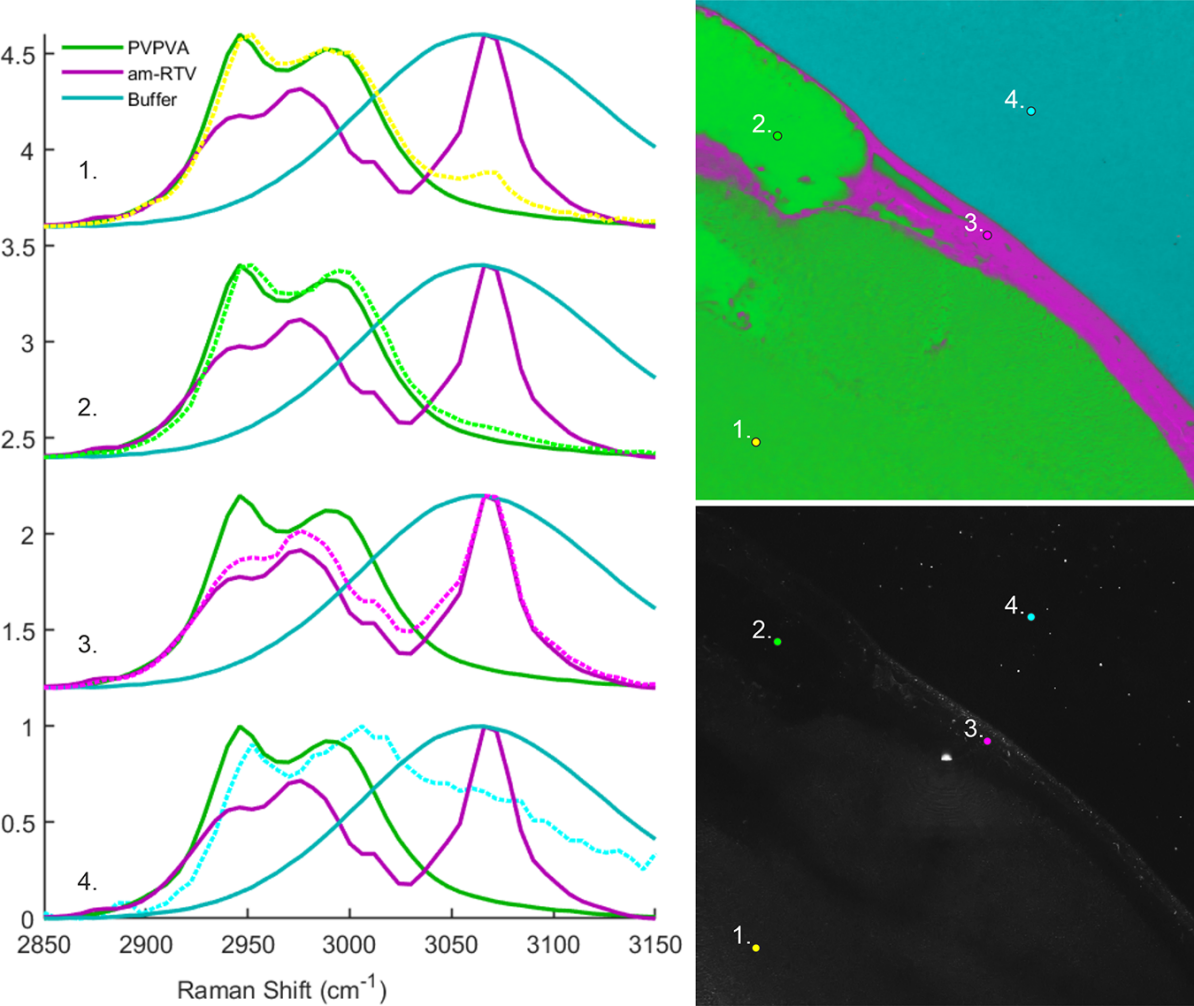
Example point spectra of RTV-PVPVA matrix film at 20% drug loading at the end of a dissolution experiment (time point 25 min after adding the phosphate buffer solution (pH 6.8)) (left). CLS reference spectra are labeled and shown in darker colors, while point spectra are shown with dotted lines. Their positions are indicated with numbered, colored dots in the false-color SRS (top right) and correlative reflection (bottom right) images. Image size is 212 × 212 μm.

Finally, 25 min after buffer addition and the film structural changes had slowed, we recorded an additional SRS image with a high spectral resolution (Figure 10), together with the correlative reflection image. We directly compared four SRS spectra from different positions in the image, to reference SRS spectra of the amorphous RTV, PVPVA, and buffer. The spectrum (yellow) at position 1 (yellow dot) is from a point where the confocal reflection data in Figure 9 suggested that phase separation had occurred at around 5 min, but the SRS data suggested that this phase separation was below the spatial resolution of the chemically specific SRS imaging. This spectrum is most consistent with the reference PVPVA spectrum (peaks at approximately 2940 and 2990 cm^–1^), but with a minority drug signal also present, as evidenced by the smaller peak at 3070 cm^–1^. The spectrum from position 2 (bright green), representing the polymer-enriched phase, is even more polymer-rich with the loss of the amorphous RTV peak. The spectrum from position 3 (magenta) is broadly consistent with amorphous RTV, with some minority PVPVA likely also contributing to the signal. The spectrum at position 4 is from the dissolution medium. This spectrum has spectral features consistent with both the polymer and the buffer. Peak shifts relative to the reference PVPVA spectrum, consistent with hydrogen bonding between the polymer and water, are also apparent (approximately 3 cm^–1^ for positions 1 and 2 and 6 cm^–1^ for point 4) and could potentially be used as an additional characteristic to measure the hydration level of the polymer.^43^ The SRS false-color image shows a single LLPS particle in the middle-right part of the buffer-rich region. The comparatively large pixel size employed, as well as constant movement of the particles, makes them more difficult to detect with hyperspectral SRS, whereas a large number of LLPS particles (small bright dots in the buffer-rich region) are visible in the correlative reflection image.

#### 40% Drug Loading

**Figure 11:**
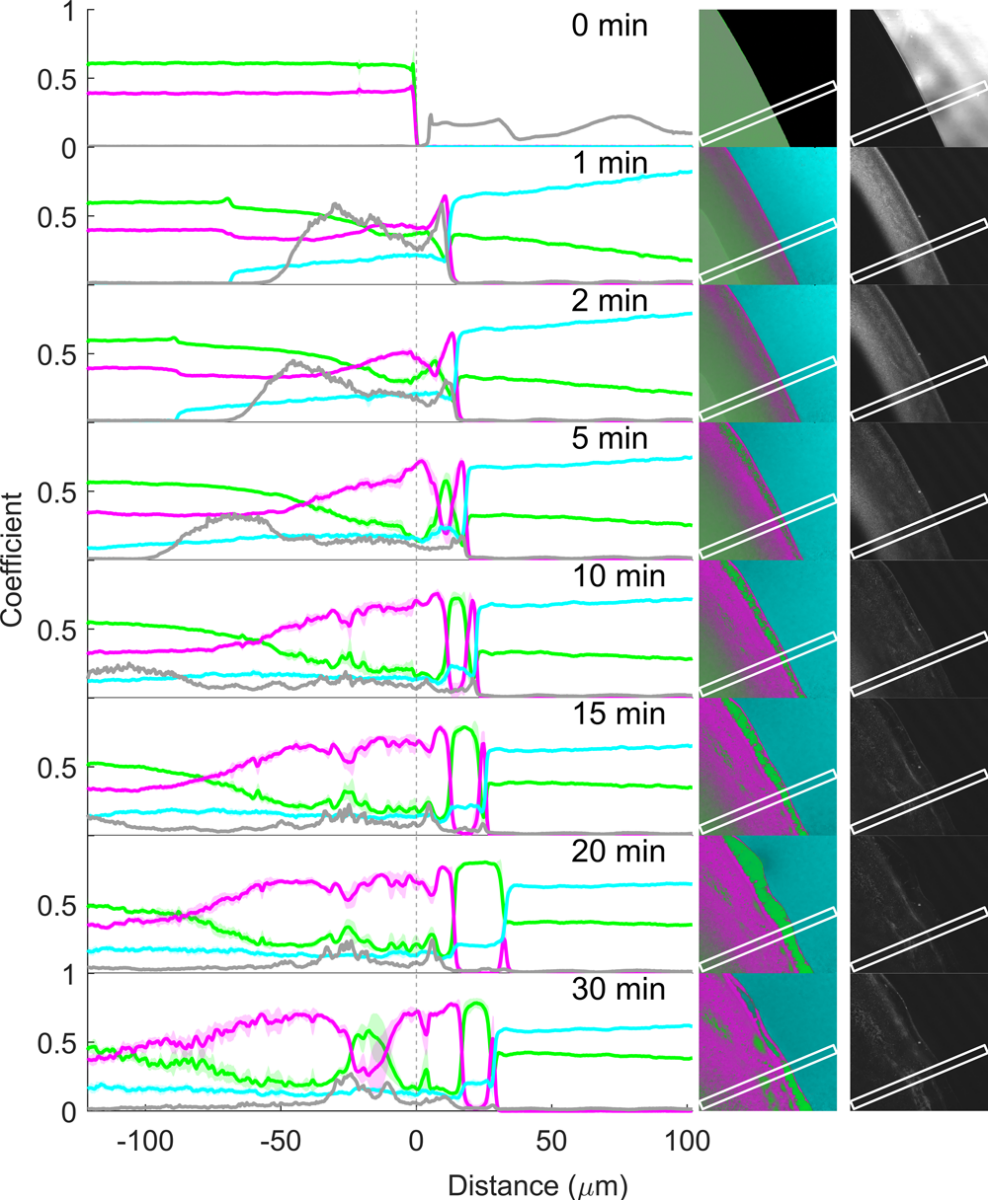
Coefficient (representing relative concentration) profiles of RTV (magenta), PVPVA (green), and pH 6.8 phosphate buffer (cyan), as well as confocal reflection intensity (gray), before (0 min) and 1, 2, 5, 10, 15, 20, and 30 min after buffer addition to matrix film with 40% drug loading. The coefficients along the x-axis are the mean (±SD for RTV, PVPVA, and buffer) at each point along the marked rectangular region in the corresponding images to the right. The vertical dotted line at 0 μm represents the film–air interface before buffer addition (0 min).

The false-color SRS images of the 40% drug loading films, together with the respective correlative confocal reflection images, are presented in Figure 11 (right), as well as the corresponding coefficient plots at each time point (left). Many of the same phenomena observed for the ASD film with 20% drug loading were also observed at 40% drug loading, but they differed quantitatively.

The false-color SRS image of the dry film (0 min) was an almost uniform shade of green, consistent with a single-phase homogeneous film richer in PVPVA than in RTV, and the film–air interface was sharp. The green color was less rich than for the 20% drug loading due to the larger contribution of the RTV signal (magenta). The SRS coefficients extracted from the rectangle in the image together with minimal standard deviations (not visible) are consistent with a homogeneous 40% drug and 60% polymer loading across the film.

Upon buffer addition, the buffer (or water) signal (cyan) is visible toward the top right-hand corner of the images. As with the 20% drug loading, the penetration of water into the matrix resulted in the glass–gel interface that is visible as a discrete change in shade of green toward the bottom left-hand corner of the SRS false-color images, as well as the appearance of the water signal in the corresponding coefficient plots. However, the water penetration was slower for the ASD with 40% drug loading than for the 20% drug loading, with the interface now appearing at approximately −68 μm and −90 μm from the original interface at 1 and 2 min, respectively (also see Figure S4). In addition, the discrete increase in the water content at this interface was slightly lower, at about 8%. At later time points, the glass–gel interface again penetrated into the film beyond the field of view. Overall, compared to the 20% drug loading, the water concentration in the film is lower, and conversely, the polymer concentration in the buffer is also lower.

Rapid drug-polymer phase separation is also clearly evident for 40% drug loading. Already within 1 min of buffer exposure, surface-directed and bulk drug-polymer phase separation has occurred, with both drug-rich (pink) and polymer-rich (green) domains.

Again, water preferentially associated with the hydrophilic polymer (as revealed by the coefficients plots). At the 40% drug loading, the magenta drug signal is more prominent across the films, and at 1 min, the drug layer at the surface is a few micrometers thicker, is more uniform, and has a higher peak drug concentration (about 70%) compared to the 20% drug loading. Once again, the phase separation intensifies over time, and surface-directed waves of alternating drug- and polymer-rich domains develop, consistent with surface-directed spinodal decomposition. At 40% drug loading, the peak drug concentrations are greater and the drug-rich domains appear more continuous also within the matrix where bulk phase separation dominates.

The correlative confocal reflection images in Figure 11 (right, grayscale) and their corresponding reflection coefficients (gray line, Figure 11 left) reveal that once again, from 1 min onward, the films again exhibit significant confocal reflection, consistent with nano- and microscale phase separation, which evolves within the films over time. Again, the reflection “wave” in the gel phase gradually progresses deeper into the gel phase of the film, albeit more slowly than for the 20% drug loading, with its onset position appearing in the gel phase at approximately −49 μm, −63 μm, and −91 μm at 1, 2, and 5 min, respectively. This onset trails behind the glass–gel interface by about 19 and 27 μm at 1 and 2 min, respectively (also see Figure S4).

Interestingly, in this particular sample, the mechanical strain on the phase-separated matrix is also evident. The polymer-rich layer behind the drug-rich surface layer continues to expand until 20 min, at which point it bursts through the drug layer, releasing most of the polymer and accompanied by a contraction of the matrix. Regardless of this mechanical disruption, overall, a significant amount of polymer was present in the buffer medium from 1 min onward, while the drug signal was not detectable. Furthermore, in contrast to the 20% drug loading, neither SRS nor confocal reflection measurements suggested the presence of any (nano)droplet formation in the buffer-rich phase. These findings are broadly consistent with the earlier observations for ASDs of the same composition.^7,10,35^

#### 60% Drug Loading

**Figure 12:**
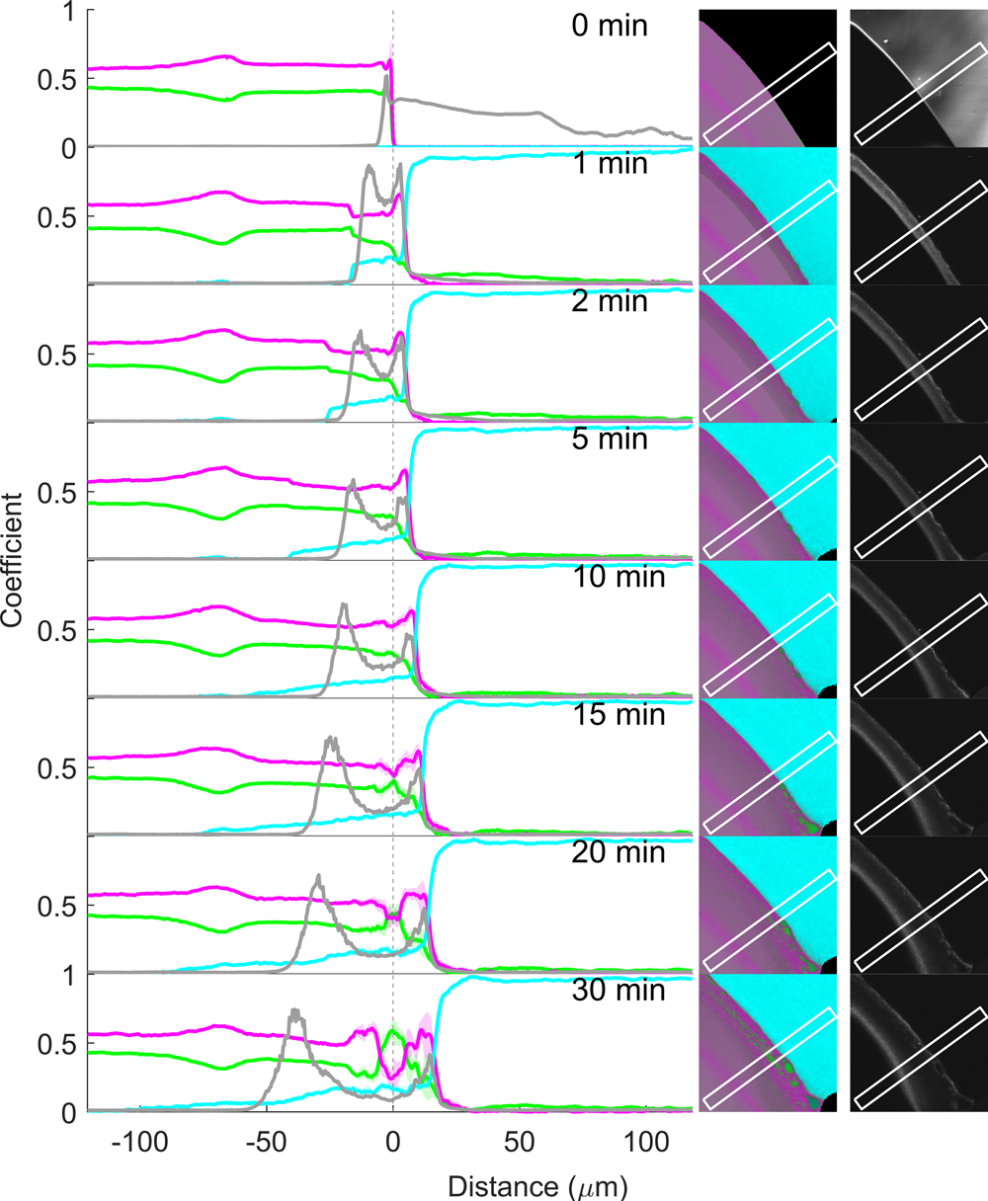
Coefficient (representing relative concentration) profiles of RTV (magenta), PVPVA (green), and pH 6.8 phosphate buffer (cyan), as well as confocal reflection intensity (gray), before (0 min) and 1, 2, 5, 10, 15, 20, and 30 min after buffer addition to matrix film with 60% drug loading. The coefficients along the x-axis are the mean (±SD for RTV, PVPVA, and buffer) at each point along the marked rectangular region in the corresponding images to the right. The vertical dotted line at 0 μm represents the film–air interface before buffer addition (0 min).

The false-color SRS image of the dry 60% drug loading film (0 min, Figure 12, right) was a largely uniform shade of pink, consistent with a single-phase homogeneous film richer in drug than polymer as well as a sharp film–air interface. The corresponding SRS CLS coefficients (Figure 12, top left), extracted from the rectangle in the corresponding false-color SRS image, are consistent with a homogeneous 60% drug and 40% polymer loading across the film (a deviation of drug and polymer content of about 8% was observed within the film as a broad band in the film, but this does not affect the overall interpretation of phase changes). After buffer addition, the false-color SRS and correlative confocal reflection images at each time point, as well as the corresponding coefficients plots, broadly reveal several of the same phase change phenomena observed in the films with the 20% and 40% drug loadings but with substantially different kinetics.

Upon buffer addition, a glass–gel interface, representing the water penetration front, is visible as a discrete change in the shade of color within the film (Figure 12, visible at 1, 2, and 5 min). The buffer coefficient values in the coefficient plots provide additional evidence of this water penetration front, also at the latter time points. The water penetration was, however, much slower for the ASD with 60% drug loading than for both the 20 and 40% drug loadings, with the penetration front appearing at approximately −17 μm, −27 μm, −42 μm, −55 μm, −76 μm, −84 μm, and −94 μm from the original interface at 1, 2, 5, 10, 15, 20, and 30 min, respectively (also see Figure S4). In addition to the slower water penetration, the buffer coefficient values suggest that the water concentration in the gel was also lower across the gel phase at all-time points, presumably due to the higher content of the hydrophobic drug. Water penetration was also associated with gradual film swelling of up to approximately 18 μm after 30 min. Interestingly, the swelling is on the same order of magnitude as the lower drug loadings and appears to be limited by the drug-enriched matrix surface at all three drug loadings.

The SRS false-color images and coefficient plots confirm that drug-polymer phase separation also occurs at 60% drug loading, though somewhat more subtly in the SRS false-color images. A continuous drug-enriched surface layer is apparent already within 1 min of buffer exposure (visible in the 1 min SRS false-color plot and SRS coefficient plot, Figure 12), accompanied by a few encapsulated polymer-rich pockets within this surface layer (1 min SRS false-color image, Figure 12). These pockets gradually increase in size over the duration of the experiment. However, unlike at the 40% drug loading, none of the imaged polymer pockets appear to break free from the continuous drug-rich network at any point. Within the film, the reflection images clearly demonstrate bulk phase separation from the 1 min time point onward and its continued propagation in the gel phase, trailing the water penetration front by approximately 3 μm at 1 min; this gap had expanded to approximately 44 μm after 30 min. The chemically specific SRS false-color images reveal that this bulk phase separation within the film is characterized by a disperse polymer-rich phase within the continuous drug-rich phase.

Within the dissolution medium, the buffer signal is strongly visible and highly consistent (with the same shade of cyan) at all-time points in the upper-right-hand region of the false-color SRS images, suggesting that the medium remains predominantly unchanged over time. Indeed, the buffer coefficient values in this region (positive values on the x-axis to the right of the matrix–dissolution medium interface) remained close to unity (representing 100%) at all times, while the polymer and drug coefficient values remained close to zero. Overall, this suggested negligible release of both the drug and the polymer at this drug loading and can be attributed to the strong continuous drug-rich network within, and especially at the surface, of the film. This network remains intact despite some buffer penetration into the film and film swelling.

#### 80% Drug Loading

**Figure 13:**
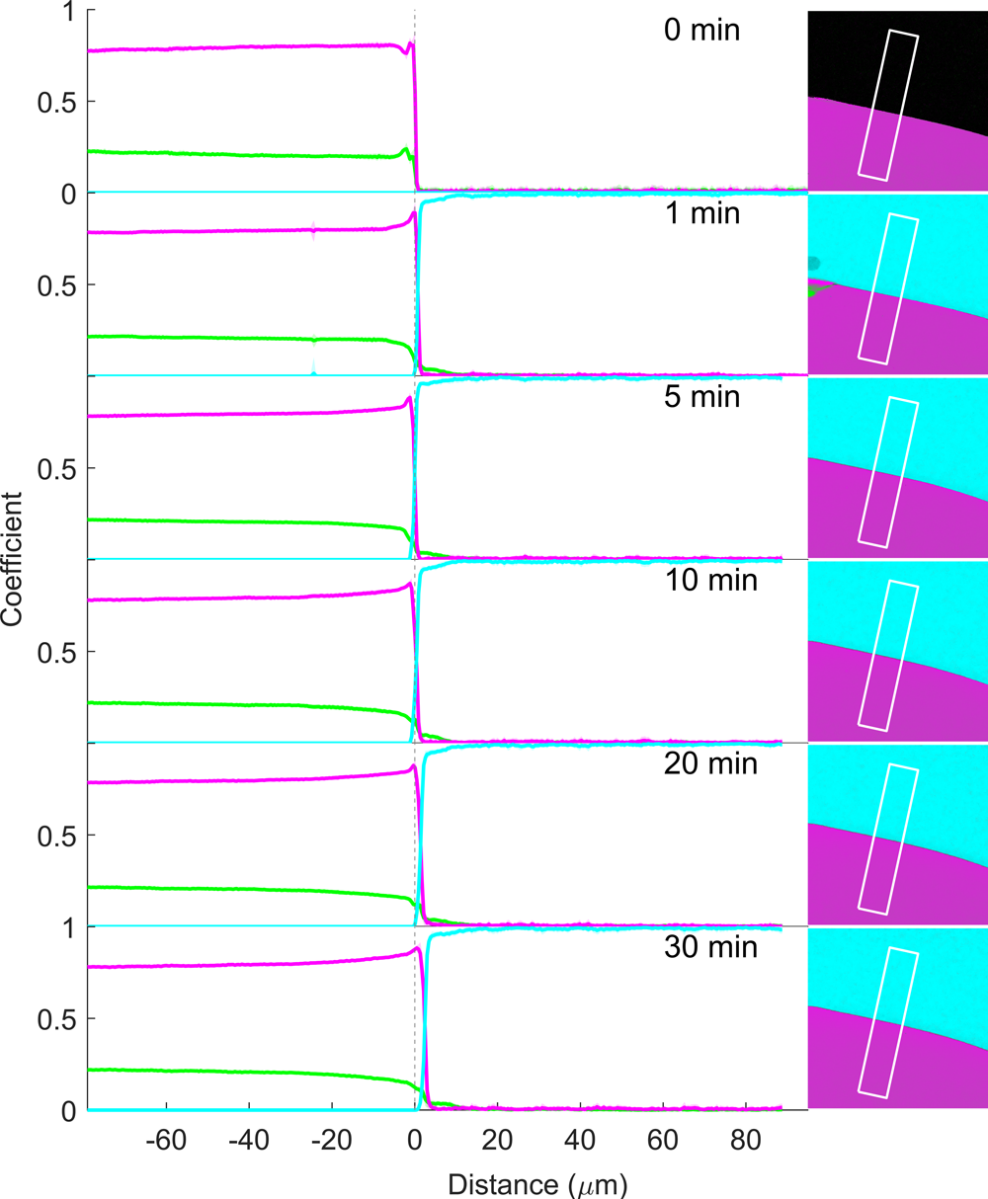
Coefficient (representing relative concentration) profiles of RTV (magenta), PVPVA (green), and pH 6.8 phosphate buffer (cyan) before (0 min) and 1, 2, 5, 10, 15, 20, and 30 min after buffer addition to matrix film with 80% drug loading. The coefficients along the x-axis are the mean (±SD for RTV, PVPVA, and buffer) at each point along the marked rectangular region in the corresponding SRS images to the right. The vertical dotted line at 0 μm represents the film–air interface before buffer addition (0 min). The sampled area was moved prior to the 5 min image due to sample burning. Due to the high degree of content homogeneity at each position along the rectangular region, the standard deviations are not visibly apparent.

The analysis of the ASD with 80% drug loading is presented in Figure 13. The false-color SRS image of the dry film (0 min) was an almost uniform shade of pink, consistent with a single-phase homogeneous film rich in ritonavir, and the film–air interface was sharp. The SRS coefficients extracted from the rectangle in the image together with minimal standard deviations (not visible) are consistent with a homogeneous 80% and 20% drug and polymer loading across the film, respectively, with a sharp film–air interface. In contrast to all the ASDs with lower drug loadings, but consistent with the pure RTV film (Figure 8), this matrix appears essentially inert with no obvious chemical or physical changes to the film for the duration of the experiment (up to 30 min), including water penetration, swelling and phase separation, and no drug or polymer release into the buffer.

#### Depth Profiling of Matrix Film

**Figure 14:**
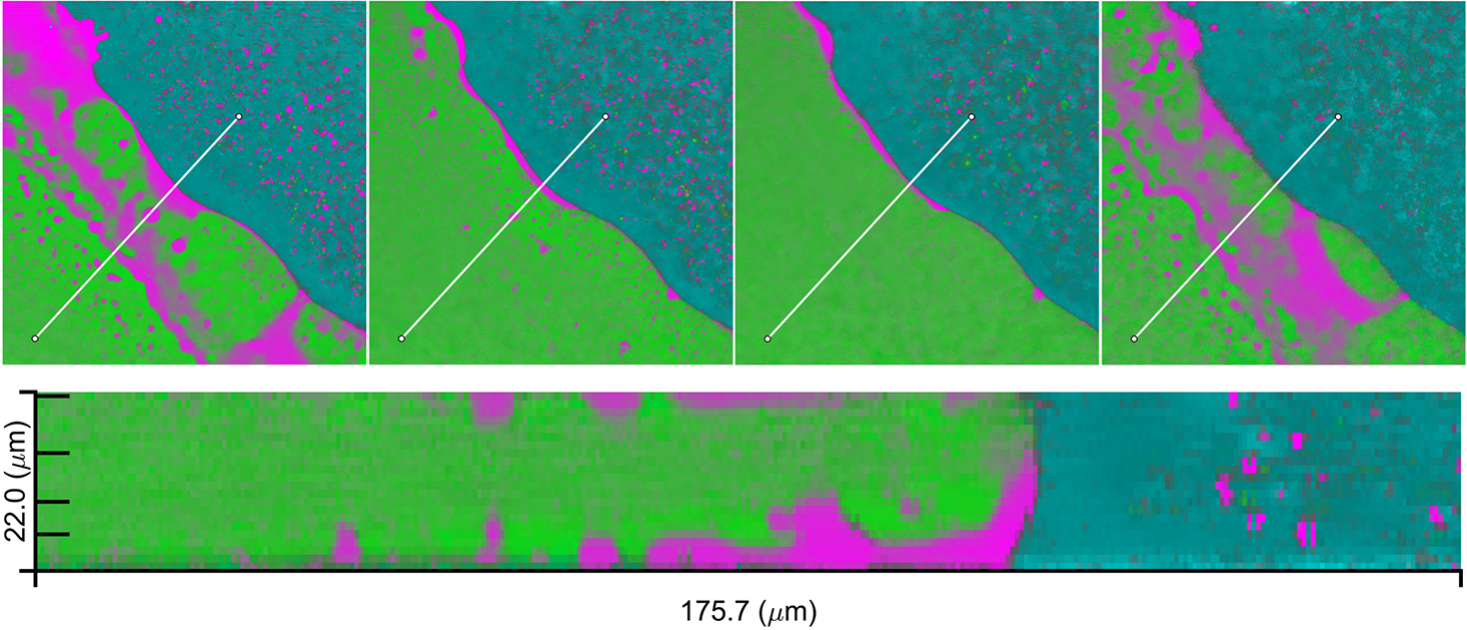
3D visualization of a matrix film at 20% drug loading (replicate experiment) past 30 min after buffer addition, with RTV (magenta), PVPVA (green), and pH 6.8 phosphate buffer (cyan). Cross-section view in the lower image for the line shown in the images above, with the images above representing normal XY plane sections. The drug-rich phase is more pronounced in the top and bottom frames, close/at the glass interfaces. The image stack height is 22 μm, and the presented images are organized from bottom (far left) to top (far right) from left to right at distances of 4, 8, 12, and 21 μm from the bottom.

We also investigated the phase separation as a function of sampling depth. For this, we recorded images at different depths over a total depth range of 22 μm within a 20% drug-loaded film (replicate experiment) at the 30 min time point (Figure 14). The images reveal significantly different distributions as a function of the sampling depth. At the top and bottom imaging depths, in the vicinity of the glass coverslips, near continuous drug-rich domains are observed, suggesting that the drug has preferentially phase-separated toward the glass during water penetration, while the polymer has concentrated toward the middle of the film. Four planes at different depths are also shown in Figure 14 (top). While phase separation is evident at each of these layers, the layers near the top and bottom (closest to the glass coverslips) show more bicontinuous drug and polymer domains across large sections of the image matrix area, while in the middle of the layer, the drug-rich phase is more predominantly represented by discrete droplets in a continuous polymer-rich phase with the continuous drug layer being restricted to the matrix–buffer interface. These substantially different observations highlight the importance of the sampling setup, and in this case in particular, being aware of the potential of glass–matrix interfaces introducing artifacts into the observed phase behavior. In our studies, we have by default imaged the film close to coverslip closest to the microscope objective to maximize spatial resolution and signal strength and therefore image quality (the detrimental effect of sampling depth on image quality is evident from the four planes shown in Figure 14). However, these effects should be kept in mind when interpreting the images with different drug loadings.

#### Limit of Congruence

In considering the congruency of drug and polymer release, our SRS (semi)quantitative imaging of the buffer-rich phase with the 20 and 40 wt % drug loadings suggests that the drug and polymer do not release congruently (i.e., proportionately), with significant polymer release and an apparent drug concentration in the buffer well below the 20:80 wt % and 40:60 wt % drug-to-polymer ratios, respectively (at 60 and 80 wt % drug loadings, neither drug nor polymer release was readily detectable).

The lack of congruent release at 20 wt % drug loading is in contrast to the results of Indulkar et al. and Yang et al.,^7,35^ who obtained congruent release for RTV-PVPVA ASDs in pH 6.8 buffer at 20% drug loading (based on HPLC analysis of the dissolution medium) and identified the limit of congruency to be approximately 25% RTV loading. However, substantial experimental and sampling differences were present between their and our setups, including compacts versus film, different dissolution setup geometries and flow dynamics (including stirring versus no stirring), and 37 °C versus room temperature. Evidence that temperature significantly alters the congruence limit already exists, as Krummnow et al.,^10^ who also investigated phase separation and release from spray-dried RTV-PVPVA ASDs at 25 °C (in stirred pH 7 dissolution medium and using UV detection), observed incongruent and disproportionately low drug release already at 20 wt % drug loading. This is consistent with our observations. More generally, we also need to emphasize that our (semi)quantitative analyses were not validated, and peak position shifts of the PVPVA signal in the film versus buffer phases (Figure 10) could have affected relative concentration predictions in the buffer-rich phase.

Our results are in line, however, with surface-oriented phase separation involving the development of the (semi)continuous drug-rich layer that we observed with *in situ* SRS imaging. Indulkar et al. and Yang et al.^7,35^ attributed their observed congruent release at 20% drug loading with the lack of the development of a continuous drug-rich layer at the film–buffer interface, which they determined using ATR-FTIR spectroscopy, SEM, and confocal fluorescence microscopy with marker dyes. Therefore, the drug release results for both their and our studies are consistent, considering the influence of the development of a drug-rich layer at the film–polymer interface. Despite this, it was interesting to note that we nevertheless still observed LLPS and nanodroplet formation at 20%, even with surface enrichment of the drug. This suggests that surface enrichment and LLPS are not necessarily mutually exclusive.

## Conclusions

In this study, we demonstrated the application of SRS microscopy, augmented with SFG and confocal reflection measurements, for label-free and chemically specific imaging of fast-paced phase changes in ASDs exposed to aqueous media. We were able to (semi)quantify the drug, polymer, and water distributions, both within the films and the dissolution medium. Phase phenomena that were imaged in real time include the water penetration front (representing the glass–gel interface), matrix swelling, dissolution, bulk, and surface-directed AAPS within the film (including surface enrichment of the drug), and LLPS within the dissolution medium. These phenomena were strongly affected by drug loading. Overall, SRS microscopy with fast spectral focusing is well-suited for qualitative and quantitative insights into water-induced ASD phase phenomena, with chemical, solid-state, temporal, and spatial resolution. These insights should facilitate ASD formulation development, especially since there is potential for quantitative imaging of more chemically complex ASDs containing multiple APIs and/or excipients.

## Supporting Information Available

Video of confocal reflection signal of from RTV-PVPVA film in pH 6.8 buffer ASD at 20% drug loading, revealing the presence and movement of nanodroplets (AVI)
Additional experimental details, materials, and methods (PDF)

## Supplementary Material

mp4c01032_si_001.avi

## Abbreviations

(AAPS): amorphous–amorphous phase separation
(ASD): amorphous solid dispersion
(CARS): coherent anti-Stokes Raman scattering
(PVPVA): copovidone
(DSC): differential scanning calorimetry
(RTV): ritonavir
(PLM): polarizing light microscopy
(SRS): stimulated Raman scattering
(SFG): sum frequency generation
(XRPD): X-ray powder diffraction
