# Supplementary material for: Dynamic Phase Behavior of Amorphous Solid Dispersions Revealed with In Situ Stimulated Raman Scattering Microscopy
Source: Mol Pharm. 2024 Nov 19;21(12):6444–57. doi: 10.1021/acs.molpharmaceut.4c01032 (PMC11615945; doi:10.1021/acs.molpharmaceut.4c01032)
Supplement: Supplementary file 2 — mp4c01032_si_002.pdf [file mp4c01032_si_002.pdf]

Supporting Information

Supporting information for manuscript “Dynamic phase behavior of amorphous solid dispersions revealed with in situ stimulated Raman scattering microscopy” by Teemu Tomberg, Ilona Hämäläinen, Clare Strachan, and Bert van Veen.

Additional details about spectral focusing

Spectral focusing is a laser pulse manipulation technique to achieve fast change of the measured Raman shift in stimulated Raman scattering (SRS) or other hyperspectral coherent Raman scattering (CRS) microscopy. In spectral focusing, spectrally broad and ideally transform limited, laser pulses (left in Figure S1) are chirped (stretched in time) such that their instantaneous wavelength (optical frequency) changes linearly over time. In this manner, the instantaneous frequency difference Ω between the Stokes and pump/probe beam, which defines the probed Raman shift, can be quickly changed by simply changing the delay Δt between the two pulses. As a result, an initial spectral resolution of 150 cm^{-1} can be reduced to 7 cm^{-1}, for example.

As a consequence of the spectral focusing and the limited pulse bandwidth, the record shape of the CRS spectrum is modified by the Stokes/pump pulse overlap function – if the pulses do not overlap in time at the sample, there cannot be any interaction generating a CRS signal. Similarly, the generated CRS signal will be the strongest when the pulses are centered on each other. This is demonstrated in Figure S1 (right) with the reference spectra of the present work. The red dotted line shows an approximation of the pulse overlap function, which multiplies the measured SRS spectra (dashed thin lines). Dividing the measured SRS spectra with the pulse overlap function results in the thick solid lines, which corresponds well with the measured spontaneous Raman spectra (thin solid lines). However, such linear scaling of spectra is usually not performed as it does not affect the analytical interpretation of the results.

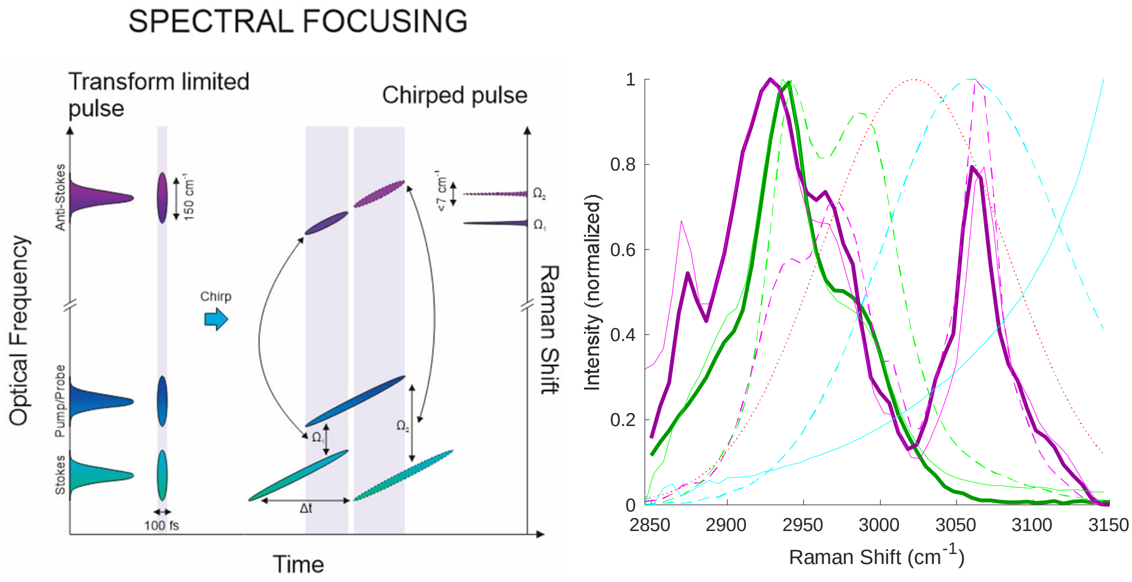

Figure S1. Illusration explaining spectral focusing spectral shape differences. On the left SRS reference spectra (dashed thin) normalization (solid thin) with gaussian shape (red dotted line) and comparison to spontaneous Raman spectra (thick lines).

Sum frequency generation microscopy of reference materials

A sum frequency generation (SFG) microscopy image of crystalline RTV is shown in Figure S2.

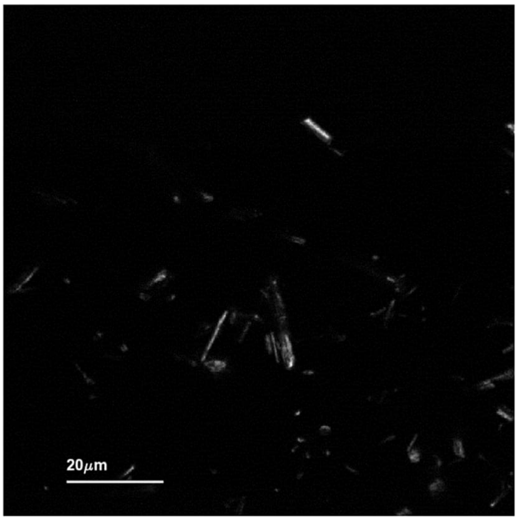

Figure S2. SFG image of crystalline RTV raw material.

Phase separation during dissolution

Ritonavir (RTV) – copovidone (PVPVA) film at 20% drug loading was the only concentration to show liquid-liquid phase separation (LLPS) particles in the buffer medium. Figure S3 shows a zoom in of the measurement in Figure 9, where LLPS are visible in the dissolution medium. The magenta strip in the bottom left corner of the images marks the matrix-buffer interface and the rest of the image to the right is the dissolution medium. Small dots are visible in the color images, with the left image showing the classical least squares (CLS) false color representation of the RTV alone, and the middle image including all three components (RTV, PVPVA, and buffer). The confocal reflection image on the right, on the other hand, shows an abundance of particles demonstrating the greater sensitivity of elastically scattered light to sub-diffraction limit particles.

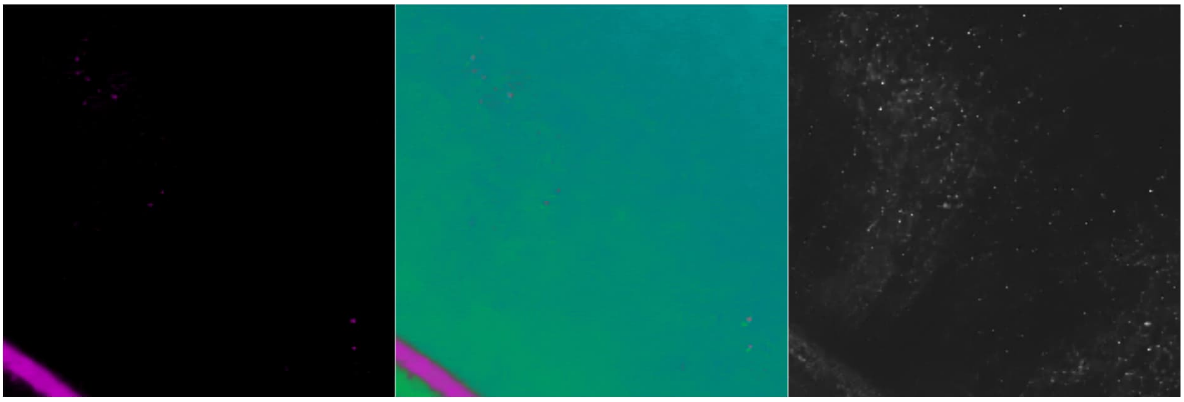

Figure S3. Example zoom in images of LLPS formation for PVPVA-RTV matrix film at 20% drug loading at 5 min time mark in the dissolution experiment. (left) CLS image of amorphous RTV alone, (middle) CLS image and (right) confocal reflection image. Largest LLPS particles are visible in the CLS image while the reflection image is most sensitive even to submicrometer particles. Ritonavir (magenta), PVPVA (green) and pH 6.8 phosphate buffer (cyan).

Film interface analysis

A separate analysis of the evolution of different film interfaces in the experiments as a function of percent drug loading is presented in Figure S4.

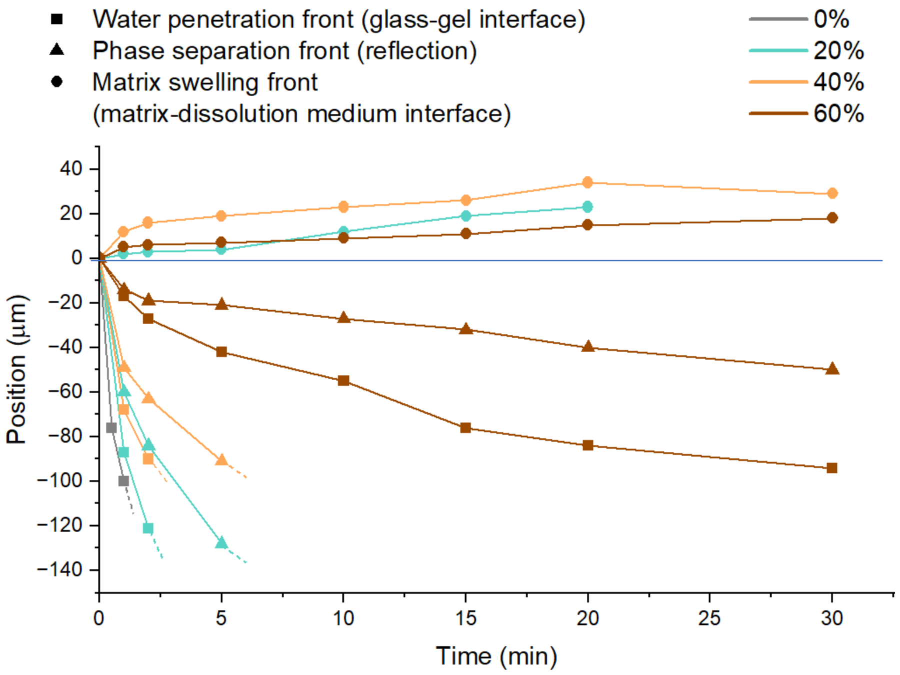

Figure S4. Propagation of film interfaces over time during exposure to buffer, relative to the initial. The film edge (film-air interface prior to buffer addition) is set at 0 Time points after adding the phosphate buffer solution (pH 6.8).

Video S1. Video of confocal reflection signal of from RTV-PVPVA film in pH 6.8 buffer ASD at 20% drug loading, revealing the presence and movement of nanodroplets.
